# Feasibility and Acceptability of Tele-Enhance Fitness in Older Adults With Knee Osteoarthritis

**DOI:** 10.1093/geroni/igab046.1091

**Published:** 2021-12-17

**Authors:** Kushang Patel, Elise Hoffman, Neta Simon, Nancy Gell

**Affiliations:** 1 University of Washington, Seattle, Washington, United States; 2 University of Vermont, Burlington, Vermont, United States

## Abstract

Enhance Fitness (EF) is an evidence-based, group exercise program for older adults. When COVID-19 halted in-person EF classes nationally, we adapted EF for remote delivery (tele-EF) by engaging key stakeholders. To determine feasibility and acceptability of tele-EF, we conducted a mixed methods study among 42 older adults (≥65 years) with knee osteoarthritis. Participants attended EF classes for 1-hour, 3 days/week for 4-5 months (1-3 months in-person EF and 2-4 months in tele-EF). Attendance for in-person EF was 80.0% versus 91.0% for tele-EF. Nearly all participants (95.2%) reported that they were satisfied or very satisfied with tele-EF. Qualitative exit interview data mapped well onto Social Cognitive Theory constructs. With tele-EF, participants found that livestream classes facilitated accountability and self-efficacy to participate in exercise and that interactive instruction provided encouragement and support to exercise. Thus, tele-EF is a viable remotely-delivered exercise program for older adults that retains many features of in-person EF.

